# Functional connectivity in distributed cortical networks associated with semantic processing in healthy older adults

**DOI:** 10.3389/fnagi.2025.1479861

**Published:** 2025-05-22

**Authors:** Kailey G. Langer, Alexandria G. O’Neal, Amanda Garcia, Alexa Chen, Eric C. Porges, John B. Williamson, Adam J. Woods, Ronald A. Cohen

**Affiliations:** ^1^Center for Cognitive Aging and Memory, College of Public Health and Health Professions, University of Florida, Gainesville, FL, United States; ^2^Department of Clinical and Health Psychology, College of Public Health and Health Professions, University of Florida, Gainesville, FL, United States; ^3^Department of Mental Health and Behavioral Sciences, James A. Haley Veterans Affairs Hospital, Tampa, FL, United States; ^4^Department of Psychology, Charles E. Schmidt College of Science, Florida Atlantic University, Boca Raton, FL, United States; ^5^Department of Psychiatry, College of Medicine, University of Florida, Gainesville, FL, United States

**Keywords:** semantics, aging, functional connectivity, dorsal attention network (DAN), semantic network (SN), default mode network (DMN)

## Abstract

**Objective:**

While word knowledge is typically well-preserved in aging, declines in executive control often observed in older adults may impact access and application of this knowledge. Evidence suggests aging is associated with declines in specialization and efficiency of pre-defined cortical networks, potentially corresponding with cognitive changes. Building upon our previous findings that delineate task-relevant semantic network activation, this study investigates connectivity patterns in additional higher-order cortical networks during a semantic association task in cognitively healthy older adults.

**Methods:**

A total of 33 older adults (61% women, 94% White, aged 70.03 ± 11.28, 16.36 ± 2.62 years of education) completed task-based functional MRI involving semantic (abstract and concrete) and phonemic (rhyming) decisions. Networks included cingulo-operculate (CON), frontal-parietal control (FPCN), default mode (DMN), dorsal attention (DAN), and a semantic network. Association between block and intra-network connectivity was assessed. If block effects were found, ROI-ROI analysis was conducted. Additionally, inter-network correlations to age were examined, along with inter-network connectivity based on task demand.

**Results:**

Age was not associated with intra-network connectivity. Semantic network connectivity increased during semantic blocks relative to rhyme. DAN and DMN connectivity increased during rhyme relative to semantic blocks, especially for abstract words. Connectivity within other networks did not differ as a function of task demands. Inter-network connectivity strength was stronger for FPCN with DAN during the rhyme blocks, and weaker during the rhyme blocks for FPCN with DMN and DMN with DAN. Older age was associated with greater inter-network connectivity for DMN with both FPCN and CON. The semantic network evidenced less intra-network connectivity during the rhyme task compared with all other networks, and greater intra-network connectivity during abstract semantic decisions compared with DAN and DMN.

**Conclusion:**

Despite trends of decreased functional specialization in aging, and prior evidence within this cohort of broad task-related activation and connectivity bilaterally, semantic task relevance remained uniquely localized to left hemisphere semantic network hubs. Increased coherence within DAN and DMN during rhyme blocks may reflect novelty of the rhyming task, underscoring flexible network recruitment for demanding tasks in healthy aging. Findings contribute to our understanding of underlying neural mechanisms involved in semantic processing in cognitive aging.

## Key points

1. Question: In normal aging, do patterns of connectivity within broad cortical networks alter when making semantic judgments about concrete or abstract words?
2. Findings: Semantic hubs were uniquely relevant to engaging in semantic judgments, and this was equally true for abstract and concrete words.
3. Importance: Findings contribute to our understanding of the underlying neural mechanisms involved in semantic processing in cognitive aging.
4. Next steps: Assess whether systems supporting semantic processing vary with word imageability as another dimension beyond concreteness.

## 1 Introduction

While the neural networks supporting semantic processing have been mapped extensively in young adults, less is known about whether these systems change with advancing age. This manuscript builds upon our prior work using this dataset (Garcia et al., 2022; Garcia et al., 2024) evaluating seed-to-voxel functional connectivity as well as whole-brain patterns of activation during semantic decision-making tasks in a population of cognitively healthy older adults. Results of the activation study found that engaging in semantic judgments corresponded with activation in areas previously established as primary hubs for semantic processing, including the left angular gyrus (AG), left middle temporal gyrus (MTG), and left inferior frontal gyrus (IFG) (Garcia et al., 2024; Hodgson et al., 2021; Meng et al., 2022). However, there also was evidence of activation extending beyond hub regions, including in the cingulate cortex, precuneus, and regions in the left frontal cortex as well as bilateral occipital cortex (Garcia et al., 2024). Additionally, activation appeared to vary to some extent as a function of the abstractness of the stimuli, where judgments about abstract word pairs (vs. concrete word pairs) appeared to elicit slightly greater bilateral activation as well as relatively greater activation of the IFG (Garcia et al., 2024). Evaluating functional connectivity, seeding from the three hub regions, resulted in further evidence of distributed processing for semantic judgments that also varied slightly depending on the abstractness of words (Garcia et al., 2022). Overall, each hub demonstrated widespread bilateral patterns of connectivity to various association areas anterior-posteriorly. Judgments about abstract concepts in particular corresponded with increased connectivity from primary semantic hubs to large clusters of voxels in bilateral frontal, parietal, occipital, and cerebellar regions (Garcia et al., 2022). Within this study, we examine the role of cortical networks beyond putative semantic hubs and their response to semantic task demands in aging adults.

For general overview, semantic cognition overall may be supported by two broad elements: (1) acquired word knowledge, including frequency of co-occurrence across concepts, social and affective experience, and multimodal perceptual experience, and (2) semantic control to effectively access, evaluate, and select lexical knowledge for appropriate use (Hoffman et al., 2018). The specific neural processes underlying engagement with semantic concepts may differ based on their level of abstractness.

Concepts with different levels of possible mental imagery, which tends to correspond with concreteness, may be more or less likely to invoke sensory-perceptual processes (Wang et al., 2010). In terms of linguistic features, including perceptual salience and affective association, words often classified as highly concrete or abstract share some overlap in characteristics but also demonstrate uniqueness along these dimensions. Abstract words tend to have more affective/social associations and concrete words tend to have greater perceptual salience (Troche et al., 2014). Neural evidence has further underscored the divergence in processing for concepts of varying abstractness, corresponding with the divergent cognitive dimensions they invoke. Regarding neural representation, some propose that concrete words may be organized by similarity in features, whereas abstract words may be organized in terms of linguistic co-occurrence and association (Montefinese, 2019). A meta-analysis identified distinct patterns of neural representation at a whole brain level across 19 studies, showing that across studies there is evidence of abstract concepts eliciting greater engagement of the verbal system, with concrete concepts eliciting greater engagement of the perceptual system (Wang et al., 2010).

Much of the current literature investigating neural correlates of semantic cognition includes healthy young adults or older adults with pathology impacting semantic function. Less is known about the functional dynamics of semantic processing within cognitively healthy older adults, who tend to maintain cognitive strengths in vocabulary but demonstrate deficits in speed of processing, cognitive control, and inhibition (Salthouse, 2019). Among adults over 50 years old, some groups have demonstrated consistency in semantic ability in aging and suggest that this stability in performance may occur alongside compensatory changes in neural activity relative to young adults (Burke and Peters, 1986; Lacombe et al., 2015). This may involve engagement of broader cortical networks associated with cognitive functions that may facilitate the control/retrieval aspect of semantic cognition, in other words the access to and application of semantic knowledge.

Results from our previous activation study in this cohort of cognitively healthy adults aged 55–85 showed that average BOLD signal was associated with accuracy of performance for both abstract and concrete semantic judgments, although within an older sample age had no association to performance accuracy or to task-related activation (Garcia et al., 2024). In the prior functional connectivity study in the same cohort, age was associated with greater semantic task-related connectivity between only the left angular and supramarginal gyri (Garcia et al., 2022). Understanding whether differential engagement from broader neural networks occurs across the age range during abstract and concrete semantic processing will be helpful for understanding whether compensatory neural activity is supporting behavioral performance, especially for abstract semantic decisions which seem to have elicited broader bilateral engagement in our prior work.

We aim to build upon our previous work and characterize patterns of functional connectivity in pre-defined cortical networks, along with a network comprised of AG, anterior MTG, and IFG as semantic hubs. Prior work in functional connectivity modeling shows that a network of these three regions underlies semantic processing (Meng et al., 2022). The other networks selected for analysis have been implicated in a range of higher-level functions that may correspond with abilities complementary to the control/retrieval aspect of semantic cognition. For example, associations have been drawn between the: cingulo-opercular network (CON) and executive function, attention, and speed (Hausman et al., 2020, 2021; Varangis et al., 2019); frontal-parietal control network (FPCN) and executive function and working memory (Hausman et al., 2021; Shaw et al., 2015); default mode network (DMN) and episodic memory, decision making, and imaginative thinking (Smallwood et al., 2021); and dorsal attention network (DAN) and working memory and distractor suppression (Lanssens et al., 2019; Majerus et al., 2018).

In the current study, we expected to observe strengthened connectivity within the semantic hub network during semantic judgments relative to the control task. Considering age was not strongly associated with either task-related activation or seed-to-voxel connectivity, we did not anticipate strong associations between connectivity and age across semantic tasks or the control task (Garcia et al., 2022, 2024). Abstract and concrete words were analyzed independently, to explore whether differences in neural engagement across higher-order cortical networks emerged. We hypothesized that, between the two task conditions, abstract word processing may associate with connectivity within networks related to cognitive control (e.g., FPCN, CON), reflecting the added component of semantic control involved in abstract word processing compared with greater reliance on perceptual imagery involved in concrete word processing. Between-network correlations and connectivity differences associated with task were also explored given evidence that this may reflect compensatory strategies or network dedifferentiation in older age. *A priori* hypotheses were not made for analyses done in an exploratory nature.

## 2 Materials and methods

### 2.1 Participants

The sample included 33 older adults (Table 1) who participated in a study of successful cognitive aging in right-handed community-dwelling older adults without evidence of cognitive impairment (Active Brain). Of 173 participants enrolled in that study, 39 were recruited with 6 participants removed from analysis for imaging quality issues or incompletion of the visit. There was no missing data that resulted in further reduction of the analytic sample size. Exclusion criteria included a prior diagnosis of neurological or major psychiatric disorder (e.g., schizophrenia), standard MRI contraindications (e.g., claustrophobia, pacemaker, other ferromagnetic body implants), mild cognitive impairment or dementia as indicated by Clinical Dementia Rating (CDR > 0), or impaired cognitive performance on the Montreal Cognitive Assessment a screening measure (MoCA < 21). Regarding the chosen MoCA cut-off, evidence indicates the typical cut-off score of 26 may be too harsh (Milani et al., 2018; Carson et al., 2018). Further, rather than relying on a single score to determine inclusion, adjudication of cognitive status was done using the preponderance data, and only participants with a CDR = 0 were included. This process was informed by the clinical experience of the principal investigator, in the interest of recruiting participants reflective of a normal aging process based on the breadth of data and avoiding unnecessary exclusions. Participants were right-handed (self-report), identified English as their primary language (self-report), and had normal language performance on neuropsychological measures presented in English (Heaton T scores > 30 on the Boston Naming Test and Controlled Oral Word Association Test). Participants provided informed consent, and all study protocols were approved by the University of Florida’s Institutional Review Board.

**TABLE 1:** Participant demographic characteristics (*n* = 33).

	Mean	Median	*SD*	Range
Age	70.03	70	11.28	43–90
MoCA total	26.73	26	2.25	22–30
Education	16.36	16	2.62	12–20

MoCA, Montreal Cognitive Assessment.

### 2.2 Stimuli

Stimuli included a subset of words for which associative norms have been developed in a group of younger adults (Troche et al., 2014). The Medical Research Council Psycholinguistic Database (MRD-PD) was used. MRD-PD is searchable by concreteness within a specified range and words given within requested ranges of concreteness were classified dichotomously as concrete or abstract (Coltheart and Evans, 1981). On a scale of 100–700, words with ratings of greater than 500 were categorized as concrete; words with ratings of less than 450 as abstract. Word pairs were created by extracting pairs of associates used by Troche et al. (2014) from the Florida Free Association norms (Nelson et al., 2004). Additional stimuli also were created by randomly combining words into pairs with similar concreteness ratings. These word pairs were then normed for level of similarity and association (on a 7-point Likert scale) by surveys administered via Amazon Mechanical Turk (Total *N* = 232). Pairs were matched for mean number of letters and written frequency for each block. All semantic stimuli had high familiarity for English-speaking adults.

To create the blocks for the dichotomous “associated/not associated” task, 30 concrete words pairs were quasi-randomly selected (10 high association, 10 low association, and 10 unassociated), and 30 abstract pairs were similarly chosen (10 high association, 10 low association, and 10 unassociated). Thirty pseudo-word pairs were then created for a rhyming block. These pseudowords were matched to the semantic stimuli for number of letters. Pseudowords were taken from a dataset of words previously used to study of non-word reading and modified for American phonetics (Besner et al., 1981). These words were all phonologically and orthographically plausible; however, they were all one syllable, so they were modified to match the longer semantic stimuli.

### 2.3 Task

**FIGURE 1:**
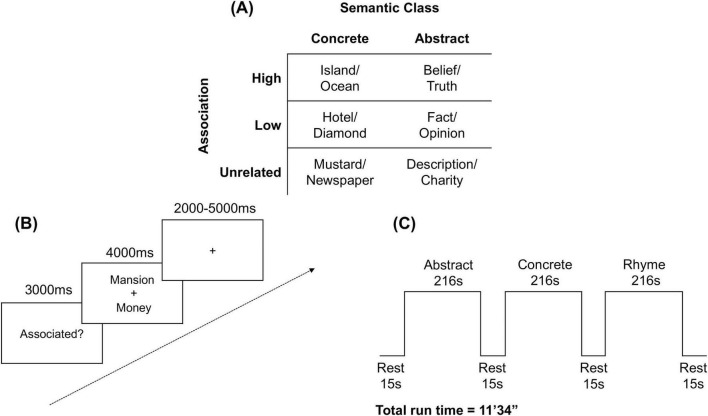
Task overview. **(A)** Examples of word pairs used; **(B)** Participant view, including task prompt, followed by a word pair eliciting a decision, followed by crosshairs; **(C)** Block design (Garcia et al., 2024).

Prior to scanning, participants completed a practice task outside the scanner with word pairs that were not used in the trials. Participants were given clarification for any potential ambiguities in the directions. For the dichotomous “associated/not associated” blocks, participants were instructed to push a button indicating whether they believed the two words were associated. They were informed that “associated” referred to whether two words were commonly used together, and they were provided with examples of words that may be dissimilar but are commonly associated (e.g., coffee, mug). Word pairs included those that share thematic association (e.g., candy, baby) or semantic taxonomy (e.g., car, boat). For the rhyming block, participants were instructed to push a button indicating whether they believed the two pseudowords rhyme (Figure 1).

Participants viewed stimuli (Arial 48 font) on a monitor behind the scanner via a mirror slotted into the head coil. Stimuli were presented using Eprime 2.0 Professional software (Psychology Software Tools, Pittsburgh, PA). Scanning was synchronized to stimulus presentation by time locking the radiofrequency (RF) pulse to the offset of each trial using a TTL synchronization box (Nordic NeuroScan, Inc.). E-prime was programmed to terminate any trial upon receipt of a signal corresponding to a new RF pulse. This procedure ensured that no cumulative timing errors were introduced into the experiment due to stimulus buffering.

Participants completed one structural and five functional sessions over the course of 1 h. Only the first three functional scans are used for analysis here. These were constructed as mixed block/event-related designs. Each scan was comprised of 3 long blocks with 30 trials each: a concrete association block, an abstract association block, and a rhyming block. The presentation order of these blocks was randomized within the run, and the presentation order of the individual trials was randomized within each block. The word pairs were identical across trials and participants were instructed to treat each trial as a new prompt, not relying on their previous answers. Participants were presented with a rest period of 15,000 ms before each block. Then a word was presented for 3,000 ms to indicate the semantic decision to be made for that block (e.g., “Associated?,” “Rhyme?”). Each word pair was presented for 4,000 ms, during which time participants pressed a button to indicate their decision. Stimulus presentation was followed by a jittered fixation cross (2–5 s). Each functional scan was 11’34” in duration.

### 2.4 Neuroimaging acquisition

Images were acquired on a Philip 3 Tesla Achieva (Amsterdam, Netherlands) with a SENSE 32-channel head coil. Foam padding was used to minimize head motion. A T1-weighted anatomical scan (TR = 7.0 ms; TE = 3.7 ms; FOV = 240 mm; FA = 8°; matrix size = 240 × 240; 170 × 1.0 mm slices) was obtained prior to functional imaging. Functional images were obtained with a 1-shot gradient echo EPI sequence (347 volumes; TR = 2,000 ms; TE = 30 ms; FOV = 224 mm; matrix size = 64 × 64; 3.5 mm × 3.5 mm in-plane resolution; flip angle = 80°). Thirty-six 3.5 mm axial slices with no gap were acquired. Preprocessing was done using fmriprep, version 1.5.4 (Esteban et al., 2017). The preprocessing pipeline applies brain extraction, segmentation, spatial normalization, and surface reconstruction to structural data and applies head motion correction, slice timing correction, and resampling into standard spaces to functional data. Functional smoothing was then performed in CONN Toolbox using a Gaussian kernel of 8 mm, followed by denoising using the default denoising pipeline (Whitfield-Gabrieli and Nieto-Castanon, 2012). The default pipeline involves ordinary least squares linear regression to estimate the following potential confounders: (1) noise from white matter and cerebrospinal fluid, (2) subject motion parameters, (3) scrubbing, and (4) session and task effects, as well as temporal band pass filtering to remove temporal frequencies below 0.008 Hz or above 0.09 Hz from the BOLD signal. Quality assurance plots within CONN were visually inspected, showing that after denoising participants’ functional connectivity distributions were approximately normal and any correlations between functional connectivity and the confounding variables listed above were eliminated. Average motion across subjects and runs was 0.07 ± 0.02 (mm). A global signal-to-noise ratio was derived by dividing the mean BOLD signal by the standard deviation in BOLD signal after preprocessing (mean = 22.59, std = 5.18, min = 10.02, max = 37.16).

### 2.5 Network parcellation

**FIGURE 2:**
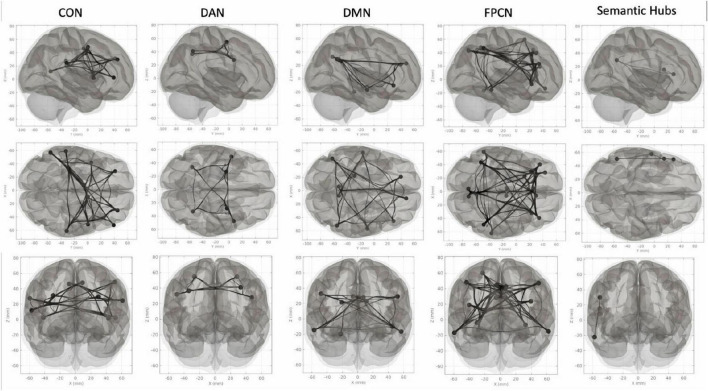
Network ROIs. From left to right: cingulo-operculate network (CON), dorsal attention network (DAN), default mode network (DMN), frontal-parietal control network (FPCN), primary semantic hubs. First row depicts a right lateral view, second row depicts an inferior view, third row depicts an anterior view.

Networks were derived using the 7-network parcellation established by Yeo et al. (2011). This atlas was applied to session-specific and subject-specific data. Within-network connectivity values for each participant [i.e., average connectivity across all network regions of interest (ROIs)] were exported from CONN Toolbox version 19c. Networks included in this study are the following: cingulo-operculate network (CON), frontal-parietal control network (FPCN), default mode network (DMN), and dorsal attention network (DAN). The remaining 3 networks were not assessed due to limited relevance to the research question and small number of ROIs and include: limbic (bilateral orbitofrontal cortex and temporal pole), visual (left lingual gyrus and right occipital fusiform gyrus), and somatomotor (bilateral precentral gyrus). Regions comprising each of these networks are reported in Table 2. Labels corresponding to ROI coordinates were taken from the Harvard-Oxford structural atlas within FSLEyes software (McCarthy, 2023). A “semantic” network was also created based on primary hub areas for semantic processing left inferior frontal gyrus opercularis and triangularis (IFG), left anterior middle temporal gyrus (aMTG), left angular gyrus (AG) and previously reported in the context of seed-to-voxel analysis in the same cohort (Garcia et al., 2022). These regions were selected from the CONN default atlas. Coordinates for ROIs in these 5 networks are presented in Figure 2.

**TABLE 2:** Network ROIs.

	Bilateral	Left	Right
CON	aSMG, COC, FP, ACC	pMTG	Pre-CG
FPCN	PRCN, FP	AG, pITG, ACC, SFG, IC, MFG	pSMG, FOC, para-CC, PCC, pMTG
DMN	pMTG, PCC	SLOC, FP, pPHG	AG, FOC, SFG
DAN	SLOC, MFG, Pre-CG	–	–
Semantic	–	IFG, aMTG, AG	–

CON, cingulo-operculate network; FPCN, frontal-parietal control network; DMN, default mode network; DAN, dorsal attention network; aSMG, anterior supramarginal gyrus; COC, central opercular cortex; FP, frontal pole; ACC, anterior cingulate cortex; pMTG, posterior middle temporal gyrus; Pre-CG, Precentral Gyrus; PRCN, Precuneous; AG, Angular Gyrus; pITG, posterior inferior temporal gyrus; SFG, superior frontal gyrus; IC, Insular cortex; MFG, middle frontal gyrus; pSMG, posterior supramarginal gyrus; FOC, frontal orbital cortex; para-CC, paracingulate cortex; PCC, posterior cingulate cortex; SLOC, superior lateral occipital cortex; pPHG, posterior parahippocampal gyrus.

Using the CONN Toolbox script, “conn_withinbetweenROItest.m,” within- and between-network connectivity values were exported for the networks of interest. This procedure results in Pearson correlations, ranging from −1 to 1. For within-network connectivity this represents an average degree of connectivity among ROIs within each network. For between-network connectivity, this represents average degree of connectivity between each set of network ROIs. We exported within-network and between-network connectivity values under each block condition (abstract, concrete, and rhyme blocks across 3 functional runs).

### 2.6 Functional connectivity

First, we evaluated functional connectivity within the 5 cortical networks of interest relative to the different tasks being performed. Connectivity values were exported from CONN Toolbox and then assessed statistically in RStudio. Each network was evaluated individually using univariate multiple regression. We assessed whether connectivity varied as a function of fMRI block, controlling for age, sex, self-reported years of education, and education-adjusted total MoCA score. We also tested an age by block interaction. All variables were *z*-scored for ease of interpretation. If significant overall block effects were observed, pairwise *t*-tests were then used to identify which blocks corresponded with significant differences in functional connectivity. Results were Bonferroni-corrected for 3 comparisons (3 fMRI blocks). Effect sizes are reported as partial eta-squared for regression and Cohen’s *d* for *t*-tests.

Considering the effects observed would indicate an overall association to functional connectivity within a given network, we were also interested in understanding whether specific ROI-ROI connections may be driving the associations observed. To investigate effects at the level of ROI-ROI connections within networks, follow-up analyses were conducted in CONN Toolbox (second-level analysis, ROI-ROI). This was only completed for networks where an overall association between block and functional connectivity was observed in the regression and *t*-test. Contrasts were Abstract > Concrete, Abstract > Rhyme, Concrete > Rhyme [−1, 1]. Contrasts were modified (i.e., Abstract > Rhyme vs. Rhyme > Abstract) based on regression results, such that the block associated with a reduction in connectivity was always coded as −1 for the corresponding network. The threshold for detecting results was set to False Discovery Rate (FDR)-corrected *p*-value < 0.05.

We investigated between-network associations using inter-network connectivity statistics exported for analysis in RStudio. In parallel to the within-network analysis, task-related fluctuations in between-network connectivity strength were explored, controlling for age, sex, education, and MoCA score. We tested the age by block interaction and used *post-hoc t*-tests to clarify any task associations identified.

Functional connectivity was not assessed relative to behavioral measures of performance in this study. Accuracy and response time both have been evaluated previously relative to block and participant age (Garcia et al., 2024). There were no associations to accuracy for age or block, or for the interaction of age by block. Response time, however, was significantly associated with both age and block. To add some behavioral context to the current findings, we did include the previously published results for response time in the following section.

## 3 Results

### 3.1 Participant characteristics

Participant demographics are described in Table 1. On self-reported medical history, all participants denied difficulty learning to read. The sample predominantly reported female sex (61%, *n* = 20) and white race/ethnicity (94%, *n* = 31). One participant reported Hispanic/Latino ethnicity and Latino race, with one other participant identifying as African American. All participants had at least high school education with most reporting a bachelor’s degree on average. As described, all participants were deemed cognitively normal based on a preponderance of evidence including MoCA score, CDR, and neuropsychological test performance. On average, however, participants still met the typical MoCA cut-off of 26.

### 3.2 Accuracy and response time

All participants showed a relatively high level of performance accuracy per prior publication in this cohort (Abstract: 79.3 + 8.4%, Concrete: 82.4 + 7.2%, Phonemic: mean = 93.5 + 5.2%) (Garcia et al., 2022). Average response time in seconds was as follows for each block (mean ± SD, range): rhyme (1.9 ± 0.26, 1.4–2.4), concrete (1.6 ± 0.20, 1.2–2.0), abstract (1.9 ± 0.32, 1.3–2.7). Response times were previously reported in Garcia et al. (2022, 2024). Published findings showed that average response time was significantly faster during the concrete semantic blocks compared with both the abstract semantic blocks (*p* < 0.001) and the rhyme blocks (*p* < 0.001). Rhyme and abstract blocks did not differ in average response time (*p* > 0.10). Older age was associated with slower response times for abstract word processing compared to concrete word processing (*p* < 0.01). Evaluating response time relative to an age by block interaction revealed that older age was significantly associated with slower response times during abstract (*p* < 0.01) and concrete (*p* < 0.01) blocks but not during rhyme blocks (*p* = 0.19). Accuracy, on the other hand, did not differ between blocks or as a function of age, and no significant age by block interactions were identified (Garcia et al., 2024).

### 3.3 Within-network functional connectivity in semantic processing

**FIGURE 3:**
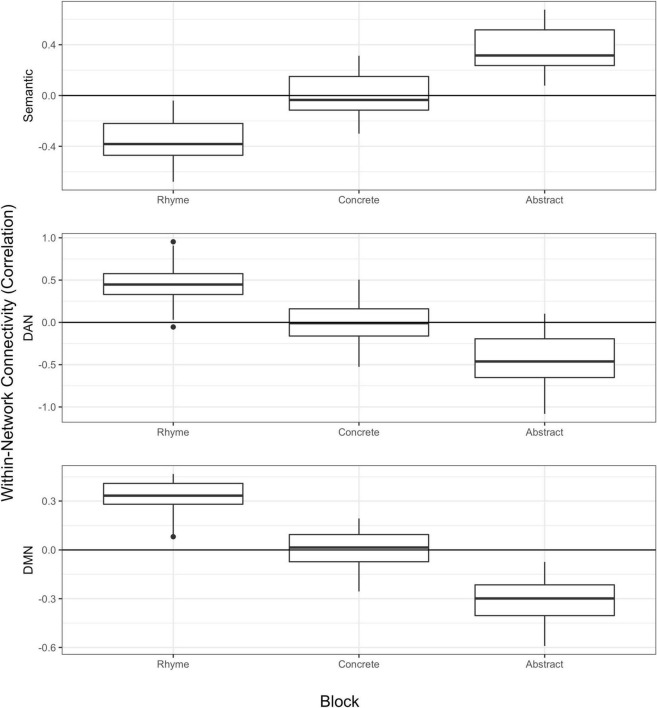
Predicted network connectivity by block. The y-axis represents predicted within-network connectivity as fitted values saved from regression including all covariates and other predictors.

Mean within-network connectivity during each block was as follows: DAN (abstract: 0.31 ± 0.13; concrete: 0.32 ± 0.12; rhyme: 0.43 ± 0.12), DMN (abstract: 0.32 ± 0.09; concrete: 0.36 ± 0.09; rhyme: 0.39 ± 0.11), FPCN (abstract: 0.22 ± 0.06; concrete: 0.22 ± 0.06; rhyme: 0.20 ± 0.07), CON (abstract: 0.26 ± 0.08; concrete: 0.27 ± 0.08; rhyme: 0.27 ± 0.07), Semantic (abstract: 0.31 ± 0.14; concrete: 0.31 ± 0.15; rhyme: 0.20 ± 0.15). All regression results showing associations between block and within-network connectivity strength are summarized in Table 3. Connectivity within semantic network ROIs was associated with block (η_*p*_^2^ = 0.09) and this is visually represented in Figure 3. *Post-hoc t*-tests were conducted to clarify block effects and revealed significantly strengthened connectivity during concrete blocks relative to rhyme [*t*(32) = −4.25, Bonferroni *p* < 0.001, *d* = 0.74] and during abstract blocks relative to rhyme [*t*(32) = −4.44, Bonferroni *p* < 0.001, *d* = 0.77]. There was no significant difference in semantic hub connectivity between the concrete and abstract semantic processing blocks [*t*(32) = 0.09, Bonferroni *p* = 1, *d* = 0.02].

**TABLE 3:** Results of within-network regression analysis.

	Semantic	FPCN	DAN	CON	DMN
Intercept	−0.22	−0.24	0.75***	0.18	0.31
Block	0.36**	0.10	−0.45***	−0.04	−0.32**
Age	0.02	−0.22	−0.03	−0.10	0.02
Education	−0.09	0.21^†^	0.08	0.27*	0.09
MoCA score	0.17	−0.02	0.15	0.25*	0.08
Sex	−0.23	0.24	−0.49*	−0.22	0.02
Block × age	−0.01	0.07	−0.07	−0.05	0.03
*R* ^2^	0.11^†^	0.06	0.21**	0.14*	0.08

DMN, Default mode network; DAN, Dorsal attention network; CON, Cingulo-opercular network; FPCN, Frontal-parietal control network. Statistics reported are standardized beta weight, partial eta-squared (reported in text), and multiple *R*-squared with *p*-values indicated for *R*^2^ and regression coefficients. *p* < 0.1^†^, *p* < 0.05*, *p* < 0.01**, *p* < 0.001***.

Connectivity within FPCN and CON did not significantly differ relative to the task being performed (η*_*p*_*^2^ < 0.01). Within DAN and DMN, there were associations such that higher degree of connectivity was observed for phonemic processing (rhyme) relative to semantic processing tasks (DAN η*_*p*_*^2^ = 0.15, DMN η*_*p*_*^2^ = 0.07). *Post-hoc t*-tests to follow-up on block effects on DAN revealed significantly higher connectivity for the rhyme block compared to both the abstract [*t*(32) = 5.50, Bonferroni *p* < 0.001, *d* = 0.96] and concrete blocks [*t*(32) = 4.53, Bonferroni *p* < 0.001, *d* = 0.94]. Abstract and concrete semantic processing were not significantly different in terms of DAN connectivity [*t*(32) = 0.50, Bonferroni *p* = 1, *d* = 0.07]. For DMN, significantly higher connectivity was observed for the rhyme block compared with the abstract block [*t*(32) = 3.73, Bonferroni *p* = 0.002, *d* = 0.63], and for the concrete block compared with abstract [*t*(32) = 2.71, Bonferroni *p* = 0.032, *d* = 0.46]. There was no significant difference in connectivity between rhyme and concrete blocks [*t*(32) = 1.30, Bonferroni *p* = 0.61, *d* = 0.23].

Age was not associated with average connectivity within any network in this sample. There also was no modifying effect of age on the association between block and intra-network functional connectivity (η*_*p*_*^2^ < 0.01). More years of education was associated with greater overall connectivity for FPCN (η*_*p*_*^2^ = 0.03) and CON (η*_*p*_*^2^ = 0.07), and higher MoCA score was associated with greater overall connectivity for CON (η*_*p*_*^2^ = 0.04). Sex was not associated with any network except DAN, where female sex was associated with lower within network connectivity on average (η*_*p*_*^2^ = 0.05).

### 3.4 ROI-ROI connectivity

Analyses were conducted in CONN Toolbox to explore specific ROI-ROI connections within networks that demonstrated overall task-related differences in functional connectivity (DMN, DAN, semantic network). The following section reviews ROI-ROI connectivity, contrasting rhyme blocks with abstract and concrete semantic blocks. Contrasts between the two semantic blocks were evaluated as well, but did not result in any significant effects using the specified criteria (FDR-corrected *p* < 0.05). This was true for all three networks evaluated. Results for contrasting abstract and rhyme blocks are presented in Table 4. In the semantic network, increased connectivity during the abstract blocks compared with rhyme was observed between IFG and each of the other network ROIs (AG, aMTG). Connectivity between AG and aMTG, however, was reduced during the abstract blocks relative to rhyme.

**TABLE 4:** ROI-ROI analysis contrasting rhyme and abstract blocks.

	Brain regions	Statistic	*p*-uncorr.	*p*-FDR
Semantic	**Cluster 1**	*F*(2, 31) = 14.11	0.00004	0.0001
	IFG oper—AG, aMTG			
	IFG tri—AG, aMTG			
	**Cluster 2**	*F*(1, 32) = 7.67	0.009	0.014
	*AG—Amtg*			
DAN	**Cluster 1**	*F*(2, 31) = 12.33	0.0001	0.0004
	Pre-CG L/R—SLOC L/R			
	**Cluster 2**	*F*(2, 31) = 12.04	0.0001	0.0004
	Pre-CG R/L—MFG R/L			
	**Cluster 3**	*F*(1, 32) = 9.97	0.003	0.006
	Pre-CG R—Pre-CG L			
	**Cluster 4**	*F*(2, 31) = 6.68	0.004	0.006
	SLOC L—MFG R/L			
	SLOC R—MFG L			
	**Cluster 5**	*F*(1, 32) = 8.34	0.007	0.008
	SLOC R—SLOC L			
DMN	**Cluster 1**	*F*(2, 31) = 21.20	0.0000	0.0000
	pMTG L—SFG R, AG R			
	pMTG R—SFG R, AG R, SLOC L			
	SLOC L—SFG R, AG R			
	FP L—SFG R, AG R			
	**Cluster 2**	*F*(1, 32) = 19.03	0.0001	0.0003
	PCC L—PCC R			
	**Cluster 3**	*F*(2, 31) = 8.32	0.001	0.002
	PCC R/L—SLOC L, FP L, pMTG L			

Semantic network contrast is Rhyme = −1, Abstract = 1; DAN and DMN network contrast is Rhyme = 1, Abstract = −1; Italics used to denote negative effects (i.e., reduced connectivity between nodes under a given contrast). DAN, Dorsal Attention Network; DMN, Default Mode Network; IFG, Inferior Frontal Gyrus; AG, Angular Gyrus; aMTG, anterior Middle Temporal Gyrus; SLOC, Superior Lateral Occipital Cortex; Pre-CG, Pre-central Gyrus; MFG, Middle Frontal Gyrus; pMTG, posterior Middle Temporal Gyrus; FP, Frontal Pole; PCC, Posterior Cingulate Cortex.

Within DAN, bilateral precentral gyri (pre-CG) evidenced stronger connectivity to one another as well as to bilateral superior lateral occipital cortices (SLOC) and bilateral middle frontal gyri (MFG) during the rhyme blocks compared with abstract. Bilateral SLOC also demonstrated stronger connectivity to one another during the rhyme blocks. Whereas left SLOC was more strongly connected with bilateral MFG during the rhyme blocks compared with abstract, right SLOC was more strongly connected with only contralateral MFG.

Within DMN, right AG as well as right superior frontal gyrus (SFG) each demonstrated strengthened connectivity with bilateral posterior middle temporal gyrus (pMTG) and contralateral frontal pole (FP) and SLOC. Left SLOC also evidenced strengthened connectivity to contralateral pMTG during rhyme compared with abstract blocks. Under this contrast, increased connectivity was observed between bilateral posterior cingulate cortex (PCC), and connecting from bilateral PCC to left hemisphere SLOC, FP, and pMTG.

Results for the concrete and rhyme condition contrasts are presented in Table 5. Within the semantic network, increased connectivity during the concrete blocks was observed between each node within IFG and each of the other network ROIs (AG, aMTG).

**TABLE 5:** ROI-ROI analysis contrasting rhyme and concrete blocks.

	Brain regions	Statistic	*p*-uncorr.	*p*-FDR
Semantic	**Cluster 1**	*F*(2, 31) = 19.18	0.0000	0.0000
	IFG oper—aMTG, AG			
	IFG tri—aMTG, AG			
DAN	**Cluster 1**	*F*(1, 32) = 27.52	0.0000	0.0001
	Pre-CG R—Pre-CG L			
	**Cluster 2**	*F*(1, 32) = 20.16	0.0001	0.0002
	SLOC R—SLOC L			
	**Cluster 3**	*F*(2, 31) = 10.93	0.0002	0.0005
	Pre-CG L—MFG R/L Pre-CG R—MFG L			
	**Cluster 4**	*F*(2, 31) = 10.50	0.0003	0.0005
	SLOC L—Pre-CG L/R SLOC R—Pre-CG L			
DMN	**Cluster 1**	*F*(1, 32) = 31.19	0.0000	0.0000
	PCC L—PCC R			
	**Cluster 2**	*F*(2, 31) = 11.71	0.0002	0.0003
	SFG R—SLOC L, FP L			
	*FOC R—FP L, AG R, pMTG L*			
	SLOC L—AG R			
	**Cluster 3**	*F*(2, 31) = 11.28	0.0002	0.0003
	SLOC L—PCC L/R			
	FP L—PCC R			

Semantic network contrast is Rhyme = −1, Concrete = 1; DAN and DMN network contrast is Rhyme = 1, Concrete = −1; Italics used to denote negative effects (i.e., reduced connectivity between nodes under a given contrast). DAN, Dorsal Attention Network; DMN, Default Mode Network; IFG, Inferior Frontal Gyrus; aMTG, anterior Middle Temporal Gyrus; AG, Angular Gyrus; Pre-CG, Pre-central Gyrus; SLOC, Superior Lateral Occipital Cortex; MFG, Middle Frontal Gyrus; PCC, Posterior Cingulate Cortex; FP, Frontal Pole; SFG, Superior Frontal Gyrus; FOC, Frontal Orbital Cortex; pMTG, posterior Middle Temporal Gyrus.

Within DAN, bilateral Pre-CG were more strongly connected to one another and to left SLOC in rhyme compared with concrete blocks. A significant increase in connectivity was also found between left Pre-CG and right SLOC. Bilateral SLOC also were more strongly connected to one another during the rhyme blocks. Bilateral MFG under this contrast demonstrated greater connectivity to left Pre-CG, with left MFG further demonstrating a significant increase in connectivity to right Pre-CG.

Within DMN, patterns of altered connectivity among specific ROIs had mixed positive and negative directionality, explaining the non-significant *t*-test comparing overall connectivity during rhyme and concrete blocks. Decreased connectivity in rhyme blocks vs. concrete was specifically observed between right frontal orbital cortex (FOC) and the following nodes: left FP, right AG, left pMTG. Bilateral PCC demonstrated increased connectivity during rhyme vs. concrete blocks to one another, as well as to left SLOC. Right PCC specifically was also more strongly connected with contralateral FP. Right AG demonstrated increased connectivity to contralateral SLOC during rhyme compared with concrete blocks. Finally, right SFG was more strongly connected with contralateral SLOC and FP.

### 3.5 Between-network connectivity

Older age was associated with greater between-network connectivity between the DMN and both FPCN (*b* = 0.33, *p* = 0.03, η*_*p*_*^2^ = 0.15) and CON (*b* = 0.39, *p* = 0.01, η*_*p*_*^2^ = 0.12), but was not significantly associated with between-network connectivity for any other network pairs (*p* > 0.1). Between network connectivity for CON and DAN was significantly, positively associated with MoCA score (*b* = 0.38, *p* = 0.001, η*_*p*_*^2^ = 0.07) and demonstrated sex differences such that women tended to show greater network segregation (*b* = −0.50, *p* = 0.04, η*_*p*_*^2^ = 0.05). Otherwise, none of the covariates included were significantly associated with between-network connectivity values (*p* > 0.05).

Average between-network connectivity by block is described in Table 6. There were three network pairings, (FPCN and DAN, FPCN and DMN, and DMN and DAN), that demonstrated significant task-related differences in between-network connectivity strength. Consistent with the within-network analyses, there were no significant differences between the two semantic task conditions (*p* > 0.1), but the between-network connectivity observed during rhyme blocks significantly differed from both semantic tasks. Relative to the rhyme blocks, FPCN and DAN were *less* strongly correlated during the concrete [*t*(32) = 2.67, Bonferroni *p* = 0.04, *d* = 0.73] and abstract semantic blocks [*t*(32) = 4.28, Bonferroni *p* < 0.001, *d* = 0.60]. Relative to rhyme blocks, FPCN and DMN were *more* strongly correlated during concrete [*t*(32) = −3.43, Bonferroni *p* < 0.001, *d* = 0.91] and abstract semantic blocks [*t*(32) = −5.15, Bonferroni *p* < 0.001, *d* = 0.71]. DAN and DMN were more strongly negatively correlated during the rhyme blocks compared to concrete [t(32) = −5.16, Bonferroni *p* < 0.001, *d* = 1.06] and abstract semantic blocks [*t*(32) = −5.79, Bonferroni *p* < 0.001, *d* = 1.11]. Across blocks, mean between-network connectivity values were positive for the FPCN with both the DAN and DMN, whereas mean values were consistently negative between the DAN and DMN. Age did not significantly moderate the association between task and between-network connectivity. No other network pairings demonstrated task-related fluctuations in connectivity.

**TABLE 6:** Descriptive statistics for between-network functional connectivity by block.

	Rhyme	Concrete	Abstract
Semantic—DAN	0.04 (0.10), −0.17–0.32	0 (0.11), −0.24–0.26	0.02 (0.09), −0.13–0.23
Semantic—DMN	0.23 (0.08), 0.09–0.47	0.27 (0.08), 0.11–0.46	0.22 (0.08), 0.08–0.45
Semantic—CON	0.03 (0.08), −0.11–0.15	0.02 (0.10), −0.20–0.19	0 (0.08), −0.17–0.17
Semantic—FPCN	0.13 (0.08), −0.03–0.3	0.15 (0.08), 0–0.32	0.13 (0.07), 0–0.29
FPCN—DAN	0.15 (0.07), −0.05–0.30	0.10 (0.07), −0.06–0.27	0.11 (0.06), 0.01–0.21
FPCN—DMN	0.09 (0.06), −0.03–0.25	0.15 (0.07), 0.01–0.33	0.14 (0.06), 0.05–0.29
FPCN—CON	0.05 (0.09), −0.12–0.25	0.04 (0.08), −0.12–0.22	0.04 (0.07), −0.08–0.18
DAN—CON	0.12 (0.07), −0.06–0.25	0.13 (0.06), 0–0.29	0.14 (0.07), −0.01–0.30
DAN—DMN	−0.17 (0.09), −0.32–0.01	−0.08 (0.08), −0.25–0.17	−0.07 (0.08), −0.30–0.08
DMN—CON	0 (0.09), −0.20–0.19	0 (0.10), −0.27–0.18	0.01 (0.06), −0.14–0.14

Values represent mean (standard deviation), min-max. CON, Cingulo-opercular Network; DMN, Default Mode Network; FPCN, Frontal-Parietal Control Network.

## 4 Discussion

The current study aimed to characterize patterns of functional connectivity in broad, higher-order neural networks during semantic and phonemic decision-making within a sample of cognitively healthy older adults. This is building on published seed-to-voxel findings showing multiple distributed “spokes” branching from semantic hubs (AG, aMTG, IFG) (Garcia et al., 2022), as well as published whole-brain activation findings demonstrating widespread bilateral activation during semantic processing tasks relative to a control condition (pseudoword rhyming) (Garcia et al., 2024). We evaluated semantic judgments for abstract and concrete word pairs, testing the hypothesis that judgments about abstract word pairs may rely more heavily on the control/retrieval aspects of semantic cognition than judgments about concrete word pairs. We sought to evaluate intra-network coherence within and beyond the semantic hubs during semantic task performance, focusing on cortical networks. The networks evaluated here were initially defined at rest, but intra-network coherence within the 4 networks selected for analyses has been shown to correspond with cognition across several domains of functioning including processing speed, attention, working memory, episodic memory, decision making, and other executive functions (Hausman et al., 2020, 2021; Lanssens et al., 2019; Shaw et al., 2015; Smallwood et al., 2021). Results demonstrated variable engagement in the DAN, DMN, and semantic network as a function of task demand. Among these three networks, we only observed increased connectivity within the semantic network during semantic tasks. These findings affirm the unique and critical role of primary semantic hubs in processing abstract and concrete words within the context of cognitive aging, as demonstrated previously (Meng et al., 2022).

### 4.1 Intra- and inter-network functional connectivity

Within the semantic network, increased functional connectivity was observed during semantic task conditions (abstract, concrete) relative to the rhyme condition, consistent with prior evidence suggesting that functional connectivity among IFG, aMTG, and AG is a reliable neural correlate of semantic cognition (Meng et al., 2022). Abstract and concrete word pairs did not elicit substantially different connectivity among semantic hubs despite the potential for abstract word pairs to represent more semantically complex concepts. Further, between-network analyses did not identify any significant task-related increase in functional connectivity between the semantic network and neural networks broadly involved in cognitive control. Across other networks we failed to identify task-related increases in within-network coherence, indicating a lack of strong evidence of vast differences in neural processing for decisions around more embodied concrete words vs. abstract word pairs with less sensory-perceptual context. In our previous seed-to-voxel study, abstract and concrete semantic conditions elicited similar functional connectivity, though there was significantly greater functional connectivity for left AG and aMTG with contralateral frontal and parietal regions during the abstract task (Garcia et al., 2022). In the present study, however, we lack strong supporting evidence for the theory that semantic decisions about abstract concepts would rely more strongly on the executive control/retrieval aspects of semantic processing than those about concrete concepts. This is consistent with our findings from a whole-brain activation study, where contrasting abstract and concrete conditions did not reveal any significant differences (Garcia et al., 2024).

Connectivity within the FPCN and CON did not differ across semantic and rhyme conditions, and in fact appeared to demonstrate stability in functional connectivity across tasks. The FPCN is primarily responsible for domain-general executive control, while the CON is associated with monitoring performance and task-irrelevant information (Dixon et al., 2018; Hausman et al., 2021; Wallis et al., 2015). The absence of task-specific effects on FPCN connectivity may be attributed to the FPCN’s involvement in overarching goal-oriented activities that applied universally across semantic and rhyme conditions. Alternatively, the study’s binary association task may have lacked the necessary complexity to sufficiently induce alterations in connectivity within control networks which may be evidenced by the fact that FPCN and CON often demonstrated lower coherence than any of the three other networks. Importantly, given the FPCN and CON encompass a larger number of ROIs, it is also plausible that any primary effects within-network were offset by the application of multiple comparison corrections. However, the FPCN did demonstrate significant task-related differences in between-network connectivity with the DAN and DMN. Relative to both semantic task conditions, the FPCN was more correlated with the DAN and less correlated with the DMN during the phonemic task. Other task-based functional connectivity studies have identified functional dynamics among these three networks. They propose that two sub-systems within the FPCN may serve to regulate cooperative coupling between the DAN and DMN (Dixon et al., 2018; Hellyer et al., 2014; Yin et al., 2022). The DAN (focused on external processes) and DMN (focused on internal processes) typically feature a pattern of anti-correlated connectivity given their divergence in functioning, but may each facilitate aspects of specific tasks (Dixon et al., 2017; Fransson, 2005). Findings here are consistent with this idea, in that the FPCN had overall positive correlations to the DAN and DMN, and the DAN and DMN were anti-correlated yet demonstrated parallel increases in coherence during the phonemic task.

The DMN and DAN demonstrated stronger connectivity during the rhyme condition in comparison to abstract (DAN, DMN) and concrete (DAN) semantic conditions. The DAN in particular appeared to demonstrate opposite patterns of task relevance to the semantic network, both through significant negative correlation across blocks and significant differences in functional connectivity during each block individually. Both of these findings were unique to DAN, though DAN and DMN appeared to demonstrate similar degrees of functional connectivity during each of the tasks performed. The DMN is characterized by its activity during self-referential and internally directed cognitive processes in the absence of a goal-oriented task (Esposito et al., 2018; Mak et al., 2017; Wirth et al., 2011). For this reason, it is unsurprising that the DMN might be more active during the rhyme task, which likely involves a degree of self-rehearsal to anticipate pronunciation and evaluate rhyming of unfamiliar pseudowords. It is possible the DMN’s role was less relevant to the abstract semantic condition, wherein there was the greatest relative decrease in within-network coherence. Average within-DMN functional connectivity was only negative during the abstract task, and this decrease in coherence may reflect increased attentional demand and cognitive control (Cohen, 2014).

The DAN is thought to facilitate top-down selective attention and distractor suppression to prioritize relevant information processing (Lanssens et al., 2019). Multiple studies support the DAN’s involvement in linguistic tasks that require working memory or focused attention on structural aspects of language, such as discriminating prosody or sentence intonation (Kristensen et al., 2013; Osaka et al., 2007; Tong et al., 2005). A phonological task likely requires additional attention and working memory toward evaluation of rhyme judgments because of the novelty of the words, which could explain why the DAN is most active during this task. The average response time during each condition may further support this interpretation of tasks demands, including novelty, need for rehearsal, and cognitive load. Response time for the rhyming condition approximated that of abstract semantic trials, with both decision types taking significantly longer to complete relative to semantic decisions for concrete words. It is important to note that. However, recent work has demonstrated a decrease in anti-correlation of the DMN and DAN networks in the context of the normal aging process, which could explain the increased coherence in both of these networks during the rhyming task (Esposito et al., 2018).

### 4.2 ROI level findings

We investigated ROI-ROI connections within each task-relevant network to discern contributions of specific brain regions to functional connectivity changes in response to task demand. Despite poor evidence supporting differential processing of abstract and concrete decisions overall among semantic hub areas, slight differences may be represented through this more nuanced view of connectivity. Existing literature supports specific relevance of AG and aMTG for integrating symbolic and embodied multisensory associative information (Jouen et al., 2015; Lin et al., 2018). It is therefore reasonable to expect a decrease in connectivity between these two integration areas when abstract words are presented in comparison to rhyme, rather than when considering concrete words, which may have a stronger sensory-motor component.

In contrast to the semantic network, the DAN exhibited increased connectivity between relatively consistent bilateral pre-frontal and posterior regions (SLOC, pre-CG, MFG) during the rhyme condition compared to both abstract and concrete conditions. This may reflect a high degree of task relevance or may be explained by the relatively small number of ROI-ROI connections which could more easily survive correction for multiple comparisons. While the DMN revealed increased connectivity among bilateral brain regions during rhyme relative to the abstract condition, comparison of rhyme to the concrete condition yielded a mixed pattern of increased and decreased connectivity between specific ROIs. Several noteworthy patterns emerged within the DMN, including frequent cross-hemispheric activity. Contrasts consistently revealed heightened connectivity between right AG and contralateral regions (PCC, SLOC) during phonemic processing. The right AG has previously been linked with functions highly relevant to the rhyming task, including attentional orienting, inappropriate response inhibition, and imagined sounds (Seghier, 2013; Tanaka and Kirino, 2019). Indeed, it has been suggested that while bilateral AG have multiple apparent functions, left AG may be more relevant to semantics whereas right AG may be more involved in attention. The reduced connectivity observed between the right Frontal Orbital Cortex (FOC) and other nodes when contrasting rhyme and concrete word conditions implies that FOC may play a more prominent role in the processing of concrete information. Of course, all of the results discussed here only represents the act of engaging in the task and not the accuracy or efficiency of the behavioral output, both of which were broadly unrelated to variations in activation or connectivity in this cohort as reported previously (Garcia et al., 2022, 2024).

### 4.3 Relevance to aging models

It’s important to consider findings in light of the age-related reorganization of neural networks described within the literature. It is widely documented that aging is associated with decreased functional network segregation and increased integration across networks to account for local inefficiency (Chan et al., 2014; Deery et al., 2023; Hausman et al., 2020; Rafiq et al., 2022). Although age was not significantly associated with intra-network coherence among the five networks evaluated, there was an age-associated trend of functional desegregation between the DMN and the two networks involved in executive functioning and cognitive control (CON, FPCN). Age-associated desegregation has been reported previously for the DMN (He et al., 2020; Lindbergh et al., 2019). He et al. (2020) used a community detection approach to quantify both segregation and integration among distributed neural networks. They found, in addition to global trends of desegregation, that nodes from the DMN, FPCN, and visual network also demonstrated increased *integration* with older age (He et al., 2020). Though the approach here differs, our findings are consistent with other findings suggesting there may be functional reorganization in normal aging that features reduced modularity, especially between the DMN and control networks.

While some studies associate the reduced network modularity to a loss of functional specialization contributing to cognitive decline, alternative perspectives propose that this may represent a compensatory mechanism aimed at preserving cognitive functioning (Cabeza et al., 2002; Grady, 2012; Heuninckx et al., 2008; Rafiq et al., 2022). While performance accuracy in this study was high and did not vary according to task (Garcia et al., 2024), as noted above increased response time during both rhyme and abstract blocks may indicate that these decisions were each more difficult than those made during concrete blocks. Specific connectivity patterns present within our results, such as the recurring cross-hemispheric activity observed in both the DMN and DAN, may potentially reflect age-related compensatory frameworks like the Hemispheric Asymmetry Reductions in Old Age (HAROLD) hypothesis (Cabeza, 2002). To establish a compensatory process, however, would require linking increased neural activity to maintained performance where we may instead expect age-related performance deficits.

### 4.4 Study limitations

While this study offers valuable insight into the sustained and critical function of a semantic network in semantic processing for older adults, it is important to acknowledge several limitations. First and foremost, the study’s sample size was relatively small, White, and highly educated, which may limit the generalizability of the findings. Further, this study was not longitudinal and did not include a cohort of younger adults, which restricts interpretation of age effects. Accordingly, our findings do not confirm the stability of semantic networks with advanced age but rather reflect that no age-related effects were detected within the limitations of our sample size and study design. Another noteworthy limitation pertains to the networks parcellation used, which exclusively include cortical regions, potentially overlooking subcortical involvement that may vary across different task conditions. The absence of a resting condition further hinders the ability to delineate specific patterns observed during phonemic processing, limiting the comprehensive understanding of the underlying neural processes. Finally, it is worth revisiting that prior studies have emphasized the collaborative function of semantic hubs alongside broader cortical association systems (Garcia et al., 2022; Jackson et al., 2016; Meng et al., 2022; Ramage et al., 2020; Yang et al., 2015). Given that these association areas also play essential roles in various cortical networks, it would be reasonable to anticipate increased connectivity between them and the core semantic network regions during the execution of semantic tasks.

### 4.5 Future directions

In future research, it will be important to explore how improved performance relates to enhanced stability in connectivity during task engagement (Cohen, 2018). Additionally, investigating the extent to which individuals exhibit “typical” patterns in functional connectivity on resting-state fMRI and relationship to cognitive performance could provide insights into the optimal organization of functional networks for various cognitive domains. This exploration may help identify patterns that are most conducive to semantic functioning. Relatedly, future integration of behavioral performance relative to functional connectivity within and between neural networks would be necessary to provide empirical support for whether any compensatory process is involved. Furthermore, future studies can delve into the comparison of younger and older adults on tasks that vary in semantic demands. This comparative analysis will further elucidate age-related differences in semantic processing and help us better understand how cognitive abilities change over the lifespan. Another dimension of word pairs featured in the semantic task not analyzed here is the degree of association between word pairs within each block (concrete and abstract). Word pairs were deemed to have high association, low association, and no association and this was used to capture average participant accuracy in their judgments. Beyond the abstractness of words, this may represent an additional aspect of task completion relevant for patterns of connectivity.

## 5 Conclusion

In summary, this study demonstrated patterns of functional connectivity among broad, higher-order cortical networks during the processing of semantic and phonemic information in a cohort of cognitively healthy older adults. A particular focus was placed on understanding the nature of semantic processing in aging by assessing network coherence within and beyond putative semantic “hubs,” including left-hemisphere AG, aMTG, and IFG. The results revealed variable engagement in the DAN, DMN, and semantic network based on task demands. Notably, increased connectivity was exclusively noted between semantic hubs during semantic task performance, underscoring the enduring and critical role of the semantic network in older adults. These findings build upon earlier research in this cohort (Garcia et al., 2022, 2024), advancing our comprehension of the neural mechanisms underpinning semantic cognition and the integrity of the semantic system in healthy aging.

## Data Availability

The original contributions presented in this study are included in this article/supplementary material, further inquiries can be directed to the corresponding author.
